# Lithotomy

**Published:** 1872-03-01

**Authors:** William J. Johnson

**Affiliations:** Chicago


### LITHOTOMY.

BY WILLIAM J. JOHNSON, M. D., CHICAGO.

On May 30, I was requested to see John Cook, aged eleven years. He was suffering very severely from all the usual symptoms of stone in the bladder, and I was told by his parents that he had been suffering about eight years, for the past three years his sufferings had much increased. Every time he attempted to micturate (which was very often) he would cry bitterly from the pain. His screams were so loud that the neighbors a block distant would hear them. The pain was referred principally to the end of the penis. During the night he was frequently obliged to get out of bed from a desire to empty the bladder, and would cry with pain, as he did during the day. When he was about to urinate he would get on the end of his toes, and walk or run around in a circle in a stooped position with his hands on his hips or grasping the penis. In this attitude he would go around screaming with pain, frequently calling for some one of the family to help him or do something for him, at the same time his urine dribbling from him. This would generally last him half an hour, before he would get over it. For the past three months his general health had become affected, and he had almost lost his appetite. On June 5, after getting him under the influence of an anæsthetic, I sounded him, and found no difficulty in detecting the stone. I then left him some medicine to try and improve his general health before the operation. On the twenty-third of June I went to his house to operate on him, where I found him suffering with one of his awful fits.

After he got through with his fit, I placed him on the table in the usual position for Lithotomy, bound him, administered chloroform, introduced the staff, and assisted by Dr. Wait, and the boy's father I proceeded to perform the lateral operation of Lithotomy. He was very weak and nervous, and yet, it seemed to take a long time to get him thoroughly under the influence of the anæsthetic. After I had made my incision into the bladder, and was about to introduce my forceps, I discovered he was sinking. I immediately unbound him, placed him on his bed, and gave stimulants. When he recovered I left him with the intention of trying to finish the operation the following day, but when I arrived I found he was very weak and exceedingly nervous, and complaining of considerable pain in the lower part of the bowels, with suppression of urine. I probed the wound and felt the stone, but he being so nervous and excitable I could do nothing with him, so I ordered him to have an injection of warm water with a little castor oil, also tea-spoonful doses of spts. nitre dulc. every three hours until he passed water pretty freely. The third day I found him feeling much better, his bowels had moved pretty freely, and during the night the stone came from him. After the sixth day the urine came through its natural channel.

He had considerable diarrhœa with a good deal of pain, two or three times after the stone came from him, which I controlled with turpentine emulsion. It was the twenty-fifth day after the operation before the wound had entirely healed. For some time after he got well enough to go about, he had considerable difficulty in retaining his urine, for which I gave him ext. belladonna and a little later tinct. cantharides, and at the present writing he seems to enjoy perfect health.

The stone was composed of phosphate and carbonate of lime. It measured one and one-half inches long and three inches in circumference, and weighed one ounce.

